# Endurance exercise ameliorates Western diet–induced atherosclerosis through modulation of microbiota and its metabolites

**DOI:** 10.1038/s41598-022-07317-x

**Published:** 2022-03-07

**Authors:** Wen-Ching Huang, Chun-Liang Tung, Yu-Chen S. H. Yang, I-Hsuan Lin, Xin Er Ng, Yu-Tang Tung

**Affiliations:** 1 Present Address: Department of Exercise and Health Science, National Taipei University of Nursing and Health Sciences, Taipei, 112 Taiwan, ROC; 2 Department of Pathology, Ditmanson Medical Foundation Chia-Yi Christian Hospital, Chiayi, 600 Taiwan, ROC; 3 Department of Food Nutrition and Healthy Biotechnology, Asia University, Taichung, 413 Taiwan, ROC; 4 Joint Biobank, Office of Human Research, Taipei Medical University, Taipei, 110 Taiwan, ROC; 5 Present Address: Bioinformatics Core Facility, University of Manchester, Manchester, M13 9PT UK; 6 Graduate Institute of Metabolism and Obesity Sciences, Taipei Medical University, Taipei, 110 Taiwan, ROC; 7 Present Address: Graduate Institute of Biotechnology, National Chung Hsing University, Taichung, 402 Taiwan, ROC

**Keywords:** Microbiology, Physiology, Cardiology, Diseases

## Abstract

The World Health Organization determined cardiovascular disease to be the leading cause of death globally; atherosclerosis is the primary cause of the high morbidity and mortality rates. Regular physical activity is an effective strategy for maintaining endothelial health and function to prevent the development of atherosclerosis. Obesity is also a crucial risk factor for atherosclerotic progression in combination with various complications and systemic inflammation. Physiological homeostasis is modulated by the intestinal microbiota, but the mechanisms through which exercise attenuates atherosclerosis through the microbiota have not been elucidated. Therefore, we investigated the effects of endurance exercise on atherosclerosis induced by a Western diet (WD) and apolipoprotein E (ApoE) knockout in terms of microbiota parameters and metabolites. Genetically modified ApoE knockout mice (C57BL/6-*Apoe*^*em1Narl*^/Narl, ApoEKO) and wild-type mice (C57BL6/J) were divided into the following four groups (*n* = 6), namely, wild-type mice fed a chow diet (WT CD), ApoEKO mice fed a chow diet (ApoE CD), ApoEKO mice fed a WD (ApoE WD), and ApoEKO mice fed a WD and performing endurance exercise (ApoE WD EX), for a 12-week intervention. The WD significantly induced obesity and atherosclerotic syndrome in the ApoE WD group. Severe atherosclerotic lesions and arterial thickness were significantly elevated and accompanied by increases in VCAM-1, MCP-1, TNF-α, and IL-1β for immune cell chemotaxis and inflammation during atherosclerotic pathogenesis in the ApoE WD group. In addition, dysbiosis in the ApoE WD group resulted in the lowest short-chain fatty acid (SCFA) production. Endurance exercise intervention (ApoE WD EX) significantly alleviated atherosclerotic syndrome by reducing obesity, significantly inhibiting VCAM-1, MCP-1, TNF-α, and IL-1β expression, and increasing the production of SCFAs. Modulation of the microbiota associated with inflammation, such as *Desulfovibrio*, *Tyzzerella*, and Lachnospiraceae_ge, and increased SCFA production, particularly through an abundance of Rikenellaceae and *Dubosiella*, were also observed after exercise intervention. Endurance exercise can alleviate WD-induced atherosclerosis through the amelioration of obesity, inflammation, and chemotaxis signaling, which are modulated by the microbiota and derived SCFAs.

## Introduction

Cardiovascular diseases (CVDs), deleterious conditions affecting the heart or blood vessels, remain the most common cause of death globally. CVDs comprise four main types, namely, coronary heart disease, stroke or transient ischemic attack, peripheral arterial disease, and aortic disease; aortic disease is associated with fatty deposit accumulation in the arteries (atherosclerosis) and the risk of blood clots. An estimated 17.9 million deaths were attributable to CVDs in 2019, representing 32% of all global deaths. Most CVDs are highly associated with mutual risk factors including lifestyle (sedentary and physical inactivity) and diet (unhealthy diet and obesity) factors and the abuse of tobacco and alcohol^1^. Atherosclerosis is the main underlying cause of CVD, as well as the dominant cause of CVD, including myocardial infarction, heart failure, stroke and claudication. Atherosclerosis is associated with aging and inflammatory conditions and can induce acute clinical events through thrombosis or plaque rupture^2^. Atherosclerosis mainly occurs in the intima of major arteries; the bifurcation of vessels and plaque rupture often involve inflammatory cytokines, chemokines, and lipid mediators, which can be targeted therapeutically for CVD treatment^3^.

Obesity is a critical risk factor for chronic diseases, including CVD, metabolic disease, cancer, osteoarthritis, gout, and breathing problems, that can have high mortality and morbidity^4^. Obesity is typically defined using indexes such as body mass index (BMI), waist circumference, and waist-to-hip ratio. Overweight and obesity are defined as BMIs of 25–29.9 and > 30 kg/m^2^, respectively. The age-adjusted prevalence of obesity among US adults was 42.4% in 2017–2018^5^. The Taiwan Nutrition and Health Survey reported an obesity prevalence of 22.8% among Taiwanese adults from 2013 to 2016, the highest overweight prevalence in Asia^6^. Thus, obesity poses a serious challenge to public health and places a burden on health care resources. An animal model of obesity induced by a high-fat Western diet (WD) can simulate the effects of high-fat and high-calorie diets and the possible mechanisms of obesity-associated complications to assist in the development of effective strategies for obesity management^7^. Besides, obesity may be an important factor in atherosclerosis and the apolipoprotein E-deficient (Apoe knockout) mice with poor lipoprotein clearance, would be stable animal model with accumulation of cholesterol ester-enriched particles in the blood for atherosclerotic pathogenesis^8^.

A systematic review proposed that the incidence of vascular events can be reduced through adoption of a healthy lifestyle, participation in regular physical activity, weight management, and consumption of a healthy diet^9^. Modifying diet and increasing exercise could be effective strategies for obesity management. Different durations, intensities, and types of exercise exhibit varying weight loss efficacy across populations. Even without weight loss, exercise could confer health benefits by reducing the negative physiological consequences of obesity and its associated complications^10^. Furthermore, aerobic exercise promotes nitric oxide secretion, blood pressure reduction, and antioxidant enzyme induction to maintain vascular function^11^. In adults with obesity, aerobic exercise leads to greater benefits for weight control and cardiopulmonary fitness than anaerobic exercise does^12^. Thus, regular aerobic exercise, such as running or swimming, provides physiological benefits that potentially minimize the risk of obesity-associated atherosclerosis.

The gastrointestinal tract is colonized by a dynamic microbial community that demonstrates succession in relation to host age, health status, and diet. The gut microbiota plays a key role in the homeostasis of the circadian rhythm, nutritional response, metabolism, and immunity^13^. Dysbiosis may cause deleterious effects in inflammation-related diseases, such as hypertension and atherosclerosis, and microbiota-derived metabolites, such as short-chain fatty acids (SCFAs) and bile acids, could be used in therapeutic applications^14^. The microbiota can also modulate atherogenesis through the regulation of inflammation, metabolism of cholesterol, and the production of microbiota-derived metabolites^15^. Microbiota-derived SCFAs can regulate a range of physiological functions, including the maintenance of the gut barrier, glucose homeostasis, immunomodulation, and appetite regulation^16^. An intervention of endurance exercise increased gut microbial diversity and the abundance of *Firmicutes* and *Akkermansia* genera to assist in SCFA production and the amelioration of metabolic diseases^17^. In addition, a systematic review revealed that exercise could significantly modulate the microbiota to regulate oxidative stress, inflammation, metabolism, and energy expenditure^18^.

Therefore, we proposed that endurance exercise could alleviate the atherosclerosis induced by a WD and genetic deletion of apolipoprotein E (ApoE) through modulation of the microbiota and its associated metabolites.

## Methods

### Animal model

Male C57BL6/J (wild type, WT) and C57BL/6-*Apoe*^*em1Narl*^/Narl (ApoE knockout, ApoEKO) mice (8 weeks old) were purchased from Taiwan’s National Laboratory Animal Center (Taipei, Taiwan) and housed in the laboratory animal center of Taipei Medical University under barrier-maintained conditions at a constant temperature (24 °C ± 2 °C) and humidity (50–60%). To confirm the animals’ welfare, a veterinarian monitored their behavior and health status daily. After 1 week of acclimatization, the mice were allocated into the following four groups (*n* = 6, each): WT mice fed a chow diet (WT CD), ApoEKO mice fed a chow diet (ApoE CD), ApoEKO mice fed a WD (ApoE WD), and ApoEKO mice fed a WD and performing endurance exercise (ApoE WD EX). The WD (D12079B, Research Diets, New Brunswick, NJ, USA) was 17% protein, 43% carbohydrate, and 40% fat, totaling 4.67 kcal/g, and was used to induce atherosclerosis in the ApoEKO mice. The experiment, including the WD diet and exercise intervention, was conducted over a 3-month period. At study completion, the mice were anesthetized with isoflurane, and blood samples were collected from the orbital sinus for blood biochemical assessment. Transcardial perfusion was performed using phosphate buffered saline (pH 7.3) through the left ventricle after sacrifice by 95% CO2 asphyxiation. The aorta was sampled immediately and quenched in liquid nitrogen and 10% formaldehyde until further analysis.

### Endurance exercise protocol

The ApoE WD EX group underwent a 3-month endurance exercise program comprising swimming sessions performed 5 days per week in a water bath maintained at 35 °C–36 °C. The daily swimming duration was initially 30 min to allow the mice to acclimate to exercise and was increased to 40 min after 2 weeks; this intervention program was slightly modified from a similar study procedure^19^. The exercise was performed at nearly the same time daily (between 2:00 and 4:00 pm). The mice were towel-dried after swimming and placed near an infrared heater to warm up before being returned to their cages.

### Plasma biochemistry and inflammation analysis

The sampled blood was centrifuged at 3000 rpm for 10 min at 4 °C for plasma collection. Total cholesterol, triacylglycerol, low-density lipoprotein (LDL) cholesterol, and high-density lipoprotein (HDL) cholesterol were analyzed using a Modular P800 Analyzer (Roche Diagnostics, Indianapolis, IN, USA). Plasma tumor necrosis factor alpha (TNF-α) was quantified using a monoclonal antimouse enzyme-linked immunosorbent assay kit (430907, BioLegend, San Diego, CA, USA).

### Morphological analysis of atherosclerotic plaques

The proximal aorta was trimmed, and paraffin-embedded tissue samples were sectioned to a 4-μm thickness for hematoxylin and eosin (H&E) staining. Pathological examination was performed with an optical microscope (DM2700M, Leica, Wetzlar, Germany), and the atherosclerotic lesion area and aortic sinus thickness were quantified in Hamamatsu C9600-12 NDP scanning software.

### Fecal microbiota analysis

The amplification and library construction for the 16s rRNA gene were performed with reference to Illumina’s recommended protocols. The V3–V4 region of the bacterial 16s rRNA gene was amplified using the universal primers 341F (5′-CCTACGGGNGGCWGCAG-3′) and 805R (5′-GACTACHVGGGTATCTAATCC-3′) with Illumina overhang adapter sequences in the forward (5′-TCGTCGGCAGCGTCAGATGTGTATAAGAGACAG-3′) and reverse (5′-GTCTCGTGGGCTCGGAGATGTGTATAAGAGACAG-3′) primers. Then, a Nextera XT Index Kit (Illumina) was employed to adjust the Illumina sequencing adapters and dual-index barcodes of the targets in the amplicon, and aQsep100 Analyzer (BiOptic, New Taipei City, Taiwan) was used for qualification and quantification of the libraries. Finally, the libraries of indicated samples were normalized to an equimolar ratio and sequenced with an Illumina MiSeq system. The taxonomy of the inferred amplicon sequence variants (ASVs) was assigned using the SILVA ribosomal reference database (v138) with an 80% minimum bootstrap confidence threshold, and multiple sequence alignment of the ASVs was performed using DECIPHER (v2.14.0). A frequency table and taxonomy were used to form a phyloseq object for downstream bacterial community analysis with phyloseq (v1.30.0), and the UniFrac distances were calculated using the R package GUniFrac. Microbiota enrichment and difference analysis were conducted using the linear discriminant analysis effect size (LEfSe) method, and the results were visualized as a cladogram by using GraPhlAn. A linear discriminant analysis (LDA) score greater than 2 was used to identify the gut microbiota succession associated with the effects of endurance exercise on WD-induced atherosclerosis.

### Fecal SCFA analysis

Fecal SCFAs were extracted using a method described in a previous study^20^ with slight modification. In brief, fecal samples from the various treatment groups were weighed and suspended in 1 mL of water with 0.5% phosphoric acid per 0.1 g of fecal sample and stored at − 30 °C until further analysis. The samples were thawed and homogenized using an ultrasonic homogenizer (BiOptic) at a 30 W for 2 min. The homogenized samples were centrifuged at 14.8 rpm for 10 min to collect the supernatant. Then, a 300-μL aliquot of ethyl acetate was mixed into the supernatant prior to centrifugation at 14.8 rpm for 10 min. The organic phase was collected and stored at − 30 °C until further analysis.

A gas chromatography–mass spectrometry system composed of an Agilent 5977B and 7693A autoinjector (Agilent Technologies, Palo Alto, CA, USA) was used for analysis. The gas chromatograph was equipped with a Nukol™ Capillary GC Column (30 m × 0.25 mm id, 0.25 μm d_f_, Merck, Germany) with helium as the carrier gas at an injection rate of 1 mL/min. The injection volume is 1 μL and the injection temperature is 250 °C. A blank sample of hexane was arranged after five consecutive fecal sample injections to assess the residual effects. The MS detector was operated in electron impact ionization mode (electron energy, 70 eV) and scanned from 30 to 250 m/z. The temperatures of the ion source, quadrupole, and interface were 230 °C, 150 °C, and 280 °C, respectively. Identification and quantification of the SCFAs were based on the retention time of the standard compounds propionate and butyrate from the NIST 08 and Wiley 7 N libraries.

### Western blotting

The expression of the relevant aorta proteins was quantified using Western blotting. Whole protein of aorta tissues was extracted by RIPA buffer containing 10% proteinase inhibitor and protein concentrations were quantified using a BCA Protein Assay. Proteins (50 µg) was denatured by heated plate at 95 °C for 5 min and loaded on gradient percentage SDS-PAGE gels (10–12%) for gel electrophoresis. Then, the proteins were transferred to a PVDF membrane and blocked (5% nonfat milk in TBST buffer) for 1 h. Then, the primary antibodies against vascular cell adhesion protein 1 (VCAM-1; ab174279, Abcam, Cambridge, UK), monocyte chemoattractant protein-1 (MCP-1; ab214819, Abcam), interleukin 1 beta (IL-1β; 12242, Cell Signaling Technology, Danvers, MA, USA), TNF-α (11948, Cell Signaling Technology), and beta-actin (4970, Cell Signaling Technology) and horseradish peroxidase-linked secondary antibodies (antirabbit 406401 and antimouse 405306, BioLegend, San Diego, CA, USA) were applied in this study between several wash with Tris-buffered saline containing 0.1% Tween® 20 detergent. The signals of the blots were developed using an enhanced chemiluminescence (ECL) system and collected the signals using chemiluminescent image system (ChemiDoc-It 515, UVP, Upland, CA, USA)^21^.

### Statistical analysis

All data are presented as the mean ± standard error of the mean (SEM). All variables were qualified using a Kolmogorov–Smirnov test before parametric testing. Statistically significant differences in body weight, food intake, biochemical parameters, microbiota abundance, cytokine and metabolite levels, protein expression, and histological scores among the groups were analyzed using one-way analysis of variance (ANOVA) and validated with a Duncan post hoc test. SPSS v22 (IBM, Armonk, NY, USA) was used for all statistical analyses, and a significant difference was defined as one for which the probability of a type I error was < 0.05.

### Ethics approval

All experimental animal procedures were reviewed by Taipei Medical University’s Institutional Animal Care and Use Committee (IACUC), and the protocol guidelines LAC-2017-0230 were approved by the ethics committee of the IACUC. The current study was carried out in compliance with the ARRIVE guidelines.

## Results

### Effects of endurance exercise on WD-induced obesity, hyperlipidemia, and inflammation

The body weight and blood lipid profiles of the WT CD, ApoE CD, ApoE WD, and ApoE WD EX groups exhibited significant differences after 3 months (Table 1); significant differences in the initial body weight, cholesterol, and triacylglycerol were also observed among groups (F(3,20) = 4.25, *P* = 0.018; F(3,20) = 29.1, *P* < 0.0001; F(3,20) = 10.1, *P* < 0.0001, respectively) before the WD dietary and exercise intervention. The body weight in the ApoE CD group was significantly higher than those in the WT CD and ApoE WD groups before intervention, but no significant difference was noted among the WT CD, ApoE WD, and ApoE WD EX groups. The levels of the lipids cholesterol and triacylglycerol in the ApoE CD, ApoE WD, and ApoE WD EX groups were significantly higher than those in the WT CD group before intervention as a result of ApoE knockout. After 12 weeks of WD diet and exercise intervention, a significant difference was observed among the groups in body weight and cholesterol and triacylglycerol levels (F(3,20) = 36.9, *P* < 0.001; F(3,20) = 76.0, *P* < 0.0001; F(3,20) = 18.1, *P* < 0.0001, respectively). The body weight of the WD groups (ApoE WD and ApoE WD EX) was significantly higher than that of the normal chow diet groups (WT CD and ApoE CD), but the exercise significantly ameliorated the WD-induced obesity (vs. ApoE WD). The cholesterol and triglyceride levels were significantly increased in the WD group with dyslipidemia, as compared to the chow diet groups (Table 1). Dyslipidemia and inflammation, highly associated with atherosclerotic disease, were also quantified to evaluate the effects of endurance exercise. The LDL cholesterol, HDL cholesterol, and TNF-α levels differed significantly among groups after the intervention (F(3,20) = 88.8, *P* < 0.001; F(3,20) = 46.3, *P* < 0.0001; F(3,20) = 18.9, *P* < 0.0001, respectively). Compared with WT CD mice and ApoE CD, LDL, cholesterol, and triglycerides were significantly increased due to ApoE knockout, but HDL was not increased. The WD intervention induced significant increases in LDL cholesterol, HDL cholesterol, and TNF-α levels in the ApoE WD group, but exercise (ApoE WD EX) improved the HDL cholesterol and TNF-α levels.

**Table 1:** Body weight, blood lipids, and cytokine profiles.

	WT CD	ApoE CD	ApoE WD	ApoE WD EX
**Body weight (g)**				
Basal	23.1 ± 0.5^a^	24.1 ± 0.3^b^	22.5 ± 0.1^a^	23.5 ± 0.3^ab^
Final	27.7 ± 0.4^a^	26.4 ± 0.4^a^	35.0 ± 0.9^c^	30.6 ± 0.7^b^
Body weight change (%)	20.6 ± 3.6^b^	9.7 ± 2.4^a^	55.6 ± 4.7^c^	30.3 ± 2.5^b^
Food intake (g/day)	3.9 ± 0.6^b^	3.8 ± 0.3^b^	3.1 ± 0.4^a^	2.7 ± 0.3^a^
Calorie (kcal/day)	11.8 ± 0.9^a^	11.5 ± 0.9^a^	14.3 ± 1.8^b^	12.9 ± 1.4^ab^
**Blood cholesterol (mg/dL)**				
Basal	76.8 ± 2.5^a^	594.7 ± 53.8^b^	535.2 ± 31.7^b^	578.3 ± 67.2^b^
Final	74.8 ± 1.4^a^	700.8 ± 34.5^b^	1106.0 ± 126.2^c^	1445.0 ± 33.8^d^
**Blood triacylglycerol (mg/dL)**				
Basal	123.2 ± 8.9^a^	176.1 ± 9.9^b^	176.2 ± 10.3^b^	181.2 ± 3.8^b^
Final	85.3 ± 5.3^a^	127.3 ± 7.5^b^	183.8 ± 23.3^c^	209.2 ± 7.8^c^
LDL (mg/dL)	6.0 ± 0.6^a^	489.8 ± 60.0^b^	954.3 ± 111.5^c^	1277.0 ± 68.9^d^
HDL (mg/dL)	65.1 ± 1.6^a^	73.6 ± 3.4^a^	210.3 ± 40.7^b^	400.2 ± 21.3^c^
TNF-α (pg/mL)	1.2 ± 0.2^a^	1.6 ± 0.4^ab^	5.4 ± 0.6^c^	2.8 ± 0.5^b^

Values are mean ± SEM (*n* = 6). Different superscript (a, b, c, d) letters indicate significant differences (*P* < 0.05) in one-way ANOVA. Body weight changes were calculated using the ratio of the baseline weight to the final body weight.

*LDL* low-density lipoprotein, *HDL* high-density lipoprotein.

### Effects of endurance exercise on development of atherosclerotic plaques

**Figure 1:**
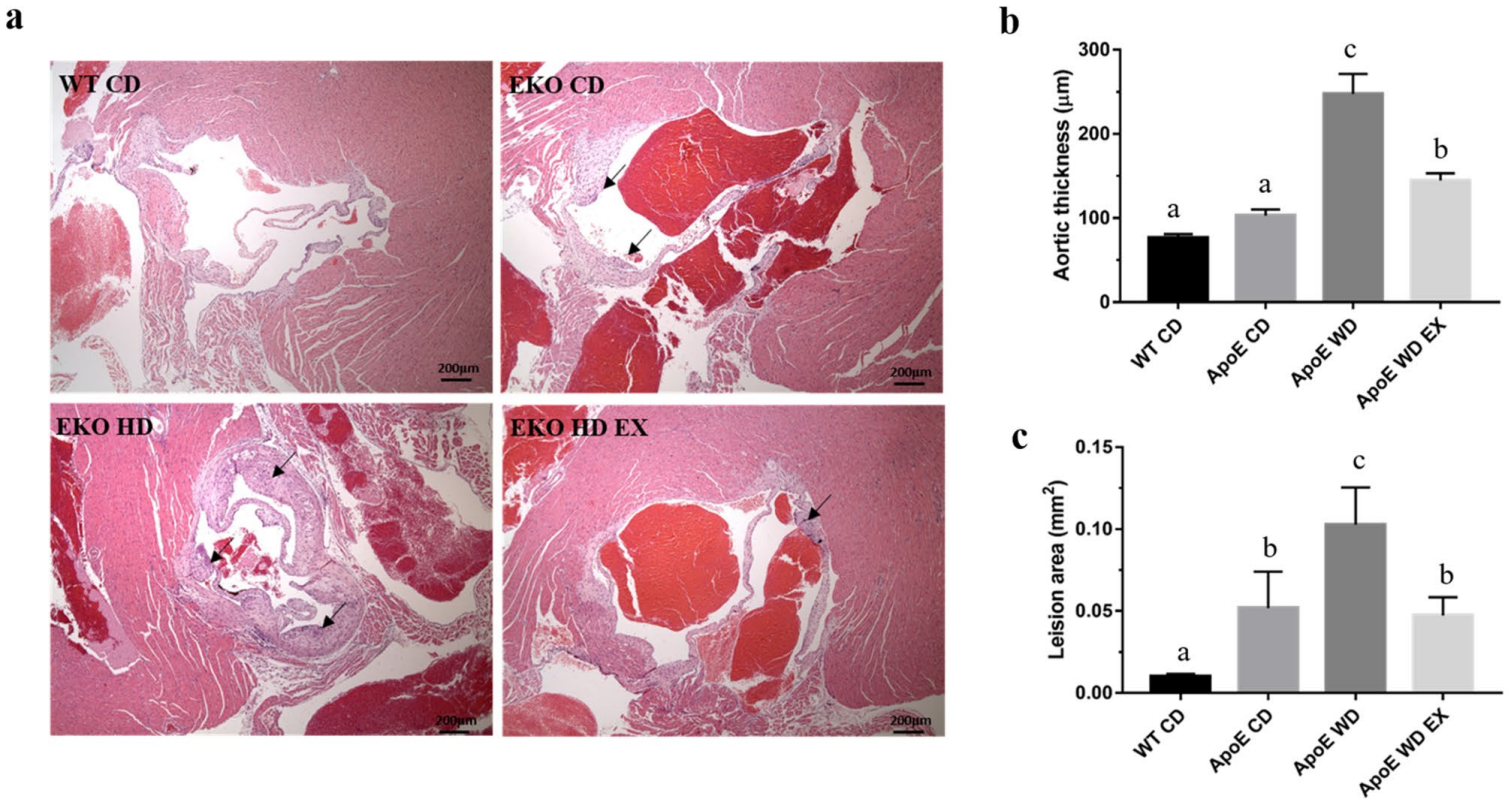
Effects of endurance exercise on atherosclerotic pathogenesis in ApoE knockout mice. (**a**) Representative H&E stain of aortic sinus sections. Arrowhead indicates atheromatous plaques. Scale bar: 200 μm. (**b**) Quantitative analysis of aortic sinus sections to detect aortic thickening and (**c**) atherosclerotic lesions. Values are mean ± SEM (*n* = 6). Different superscript letters (a, b, and c) indicate significant differences (*P* < 0.05) between groups in one-way ANOVA.

H&E staining of aortic sinus sections from the WT CD, ApoE CD, ApoE WD, and ApoE WD EX groups was used to visualize the pathological syndromes of atherosclerosis as representative photomicrographs; we quantified these images to determine vascular thickness and plaque area (Fig. 1). The arrowheads in the images of the ApoE CD, ApoE WD, and ApoE WD EX groups depicted in Fig. 1a indicates atheromatous plaques. Atherosclerotic plaques were found in the ApoE knockout mice under WD and normal chow diet conditions, and the WT CD group was regarded as a negative reference. Furthermore, WD-induced endothelium thickening in the aortic root resulted in the formation of fibrous plaques, which accelerated the development of atherosclerosis. Aortic thickness and plaque area exhibited significant differences among the groups (F(3,20) = 32.1, *P* < 0.001 and F(3,20) = 30.6, *P* < 0.0001, respectively). The ApoE knockout and WD (ApoE WD) group had significantly greater aorta thickness than the chow diet groups, but exercise (ApoE WD EX) significantly mitigated the vascular thickening caused by the ApoE knockout and WD (Fig. 1b). The ApoE knockout groups (ApoE CD, ApoE WD, and ApoE WD EX) exhibited pathological plaque with mild to severe degrees; the WD (ApoE WD) also exacerbated this pathological syndrome as compared with a normal chow diet (ApoE CD). The exercise intervention led to significantly less plaque area in the ApoE WD EX group than in the ApoE WD group. Thus, endurance exercise protects against cholesterol-driven plaque formation and vascular thickening in the aortic root during the pathogenesis of atherosclerosis.

### Endurance exercise alleviates WD-driven dysbacteriosis

**Figure 2:**
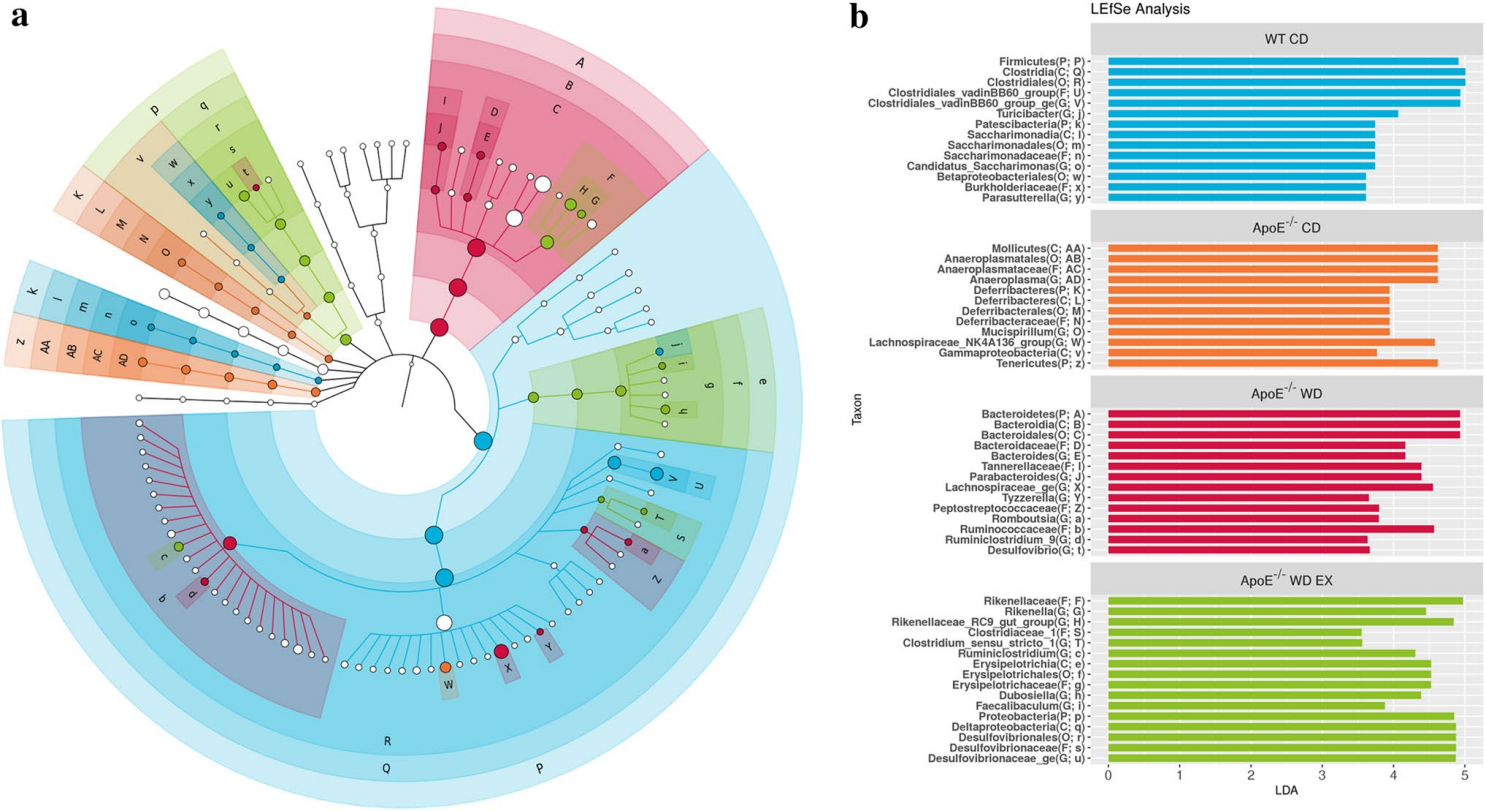
Effects of endurance exercise on gut microbiota population in ApoE knockout mice. (**a**) Cladogram depicting the phylogenetic distribution of the bacterial lineages associated with statistical differences in LEfSe analysis of the feces of different groups (**b**) LEfSe analysis of bacterial communities with LDA scores greater than 2. Differences are represented by the color of the most abundant class (light blue is WT CD; orange is ApoE CD; red is ApoE WD; green is ApoE WD EX). The abundance of each taxon is represented by each circle’s diameter in the community, with concentric circles indicating phylogenetic levels from phylum to genus.

**Figure 3:**
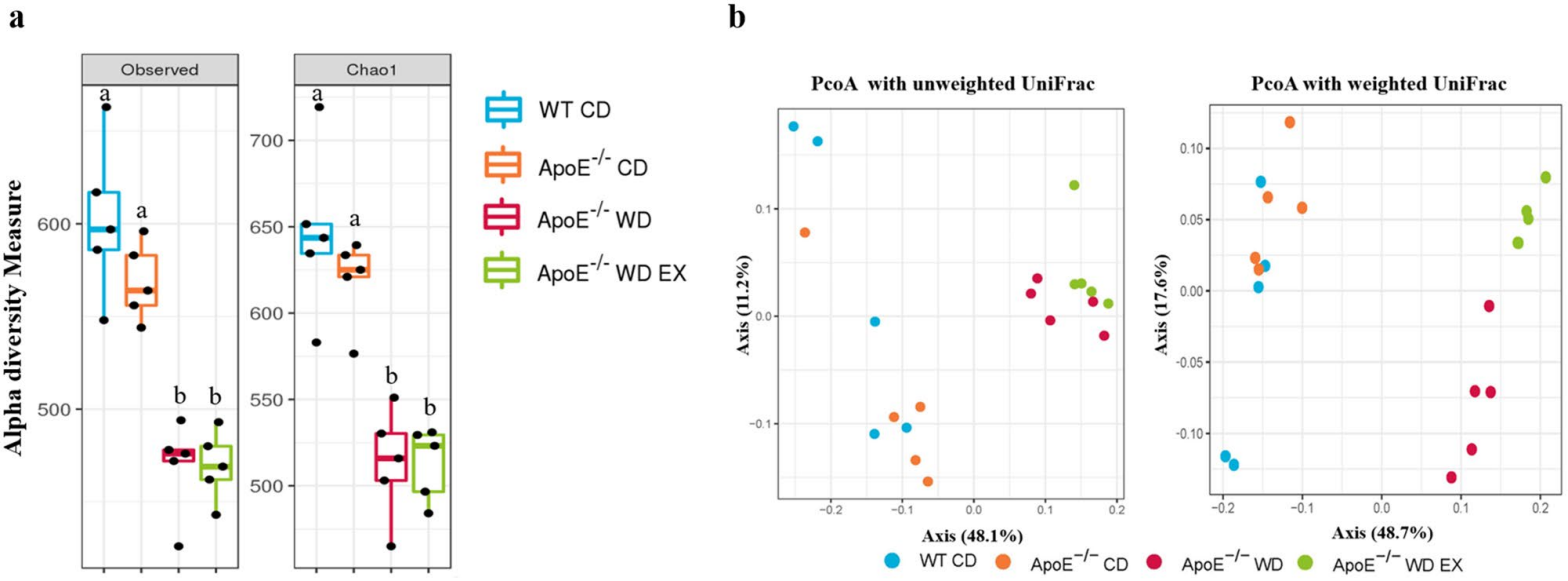
Effects of endurance exercise on gut microbiota composition in ApoE knockout mice. The (**a**) α-diversity (observed and Chao1 index) and (**b**) β-diversity (unweighted and weighted UniFrac PcoA) in the WT CD, ApoE CD, ApoE WD, and ApoE WE EX groups are indicated by the same colors used in Fig. 2. The line inside the box represents the median, and the whiskers represent the lowest and highest values within the 1.5 interquartile range. Different superscript letters (a and b) indicate significant differences (*P* < 0.05) in one-way ANOVA.

To determine the effects of endurance exercise on gut microbiota composition, we conducted Illumina-assisted 16S rRNA amplicon sequencing for 20 samples (*n* = 5 per group) to compare microbial abundance, composition, and population among the groups (Fig. 2). Intragroup and intergroup α-diversity was assessed using observation and Chao1 indexes, and β-diversity was measured through weighted and unweighted UniFrac principal coordinate analysis (PcoA). As presented in Fig. 3, the α-diversity of the WD groups (ApoE WD and ApoE WD EX) was significantly lower than that of the normal diet groups (WT CD and ApoE CD); thus, diet crucially affected intragroup microbial diversity. The distance between microbial communities (intergroup diversity) was computed from the constructed OTUs using UniFrac analysis with PcoA plots, revealing significant differences in the microbiota composition among groups (*P* = 0.001 and *P* = 0.001 in the Adonis test, respectively), with homogeneous dispersions (*P* = 0.54 and *P* = 0.41, respectively). Exercise intervention modulated the microbiota under the conditions of hyperlipidemia and a high-fat diet, acting as a physiological regulator. LEfSe and LDA assays were used to characterize the microbial communities and identify significant differences in abundance (Fig. 2); the WT CD group encompassed most of the bacterial community. In the ApoE WD EX group, the Rikenellaceae and Erysipelotrichaceae families and their associated genera, *Rikenella*, Rikenellaceae RC9, *Dubosiella*, and *Faecalibaculum*, were significantly abundant.

### Effects of exercise and WD on gut microbiota genera

**Figure 4:**
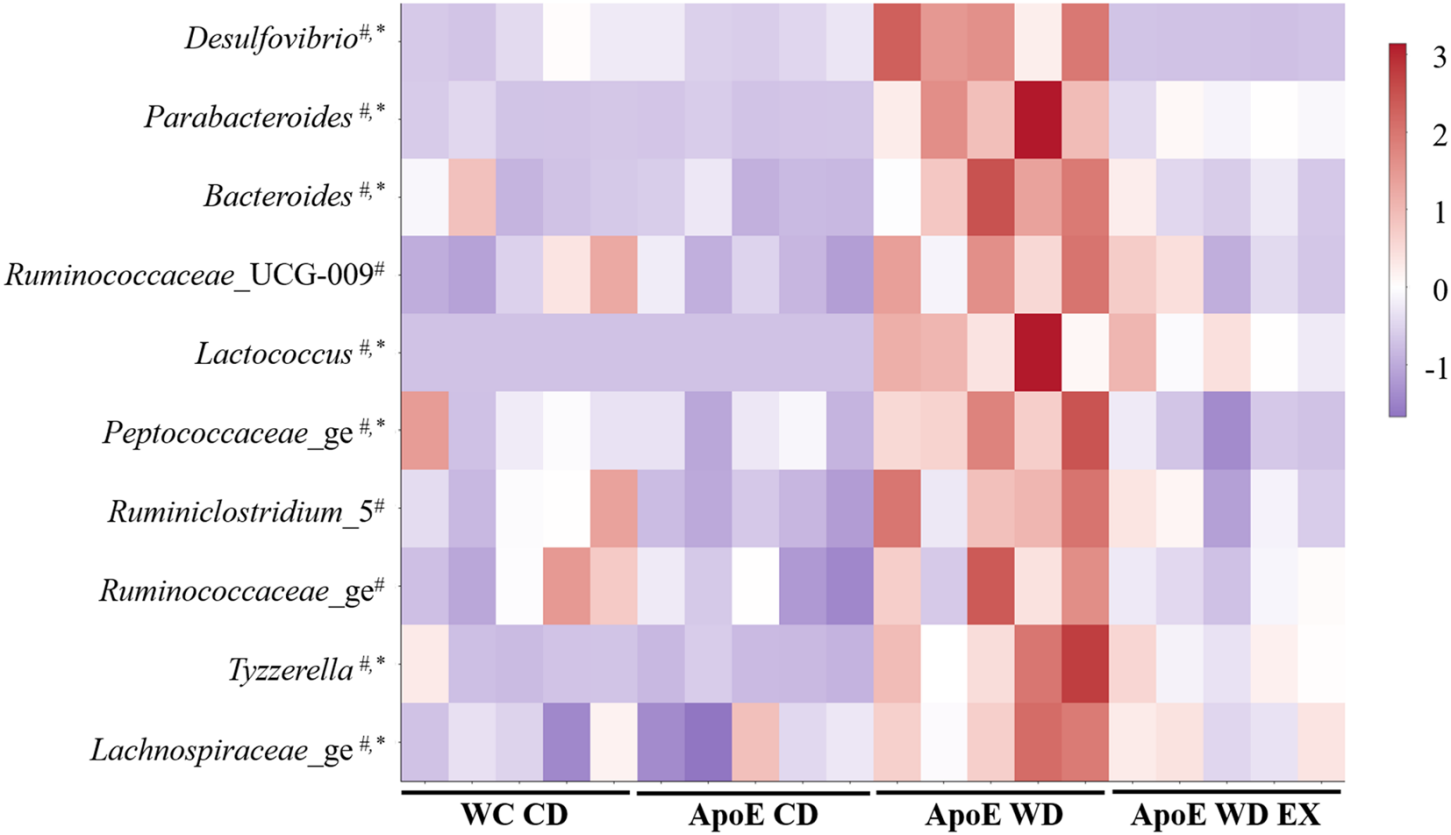
Effects of endurance exercise on gut microbiota at the genus level in ApoE knockout mice. Heatmap of integrated microbiota abundance among the WT CD, ApoE CD, ApoE WD, and ApoE HD EX groups. Significant differences were assessed using one-way ANOVA followed by a Duncan post hoc test. *Significant difference between ApoE WD and ApoE WD EX; ^#^Significant differences among ApoE WD, ApoE CD, and WT CD.

The microbial genera largely belonged to the Bacteroidetes, Firmicutes, and Proteobacteria phyla and demonstrated significant differences in abundance among groups. As described in Fig. 4, the ApoE WD group exhibited significantly higher abundance of *Desulfovibrio*, *Parabacteroides*, *Bacteroides*, Ruminococcaceae spp., *Lactococcus*, Peptococcaceae, *Ruminiclostridium*_5, Tyzzerella, and Lachnospiraceae than did the ApoE CD group. However, endurance exercise resulted in significantly lower relative abundance of *Desulfovibrio*, *Parabacteroides*, *Bacteroides*, *Lactococcus*, Peptococcaceae, *Tyzzerella*, and Lachnospiraceae in the ApoE WD EX group than in the ApoE WD group, a result consistent with the exercise-induced inhibition of atherosclerotic pathogenesis (Fig. 2). Thus, endurance exercise intervention could potentially prevent hyperlipidemia-induced dysbiosis and maintain the homeostasis of the gut microbial community for disease prevention.

### Endurance exercise promotes fecal SCFA production

**Figure 5:**
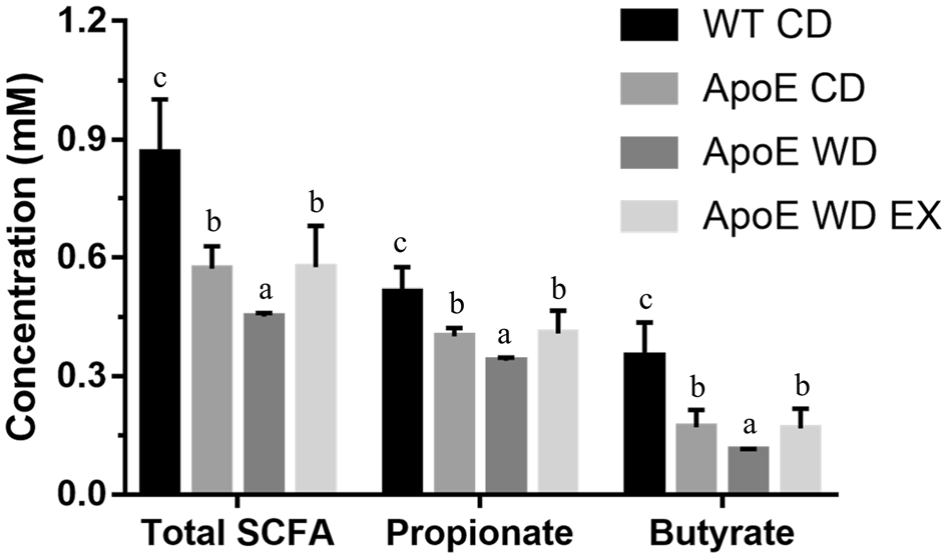
Effects of endurance exercise on fecal SCFAs in ApoE knockout mice. Values are mean ± SEM (*n* = 6). Different superscript letters (a, b, and c) indicate significant differences (*P* < 0.05) in one-way ANOVA.

CVDs and their major risk factors such as atherosclerosis and hypertension may be related by an inflammatory response highly associated with cholesterol metabolism, gut dysbiosis, and microbial metabolites^13^. Therefore, the concentration of fecal SCFAs, the main metabolites produced and fermented by microbiota from dietary fibers and resistant starch, were assessed in this study (Fig. 5). The total SCFAs and propionates and butyrate levels were significantly different among groups (F(3,20) = 22.72, *P* < 0.001; F(3,20) = 16.4, *P* < 0.0001; F(3,20) = 22.4, *P* < 0.0001, respectively). The dyslipidemia caused by ApoE knockout in the ApoE CD, ApoE WD, and ApoE WD EX groups significantly reduced the total SCFAs and propionate and butyrate levels, as compared with the WT CD group. With ApoE knockout, WD-induced hyperlipidemia (ApoE WD) resulted in significantly lower total SCFAs and propionate and butyrate levels than did the chow diet. The exercise intervention (ApoE WD EX) resulted in significantly higher levels of microbial metabolites than were observed in the ApoE WD group.

### Effects of endurance exercise on the expression of aortic and inflammatory cytokines during atherosclerosis

**Figure 6:**
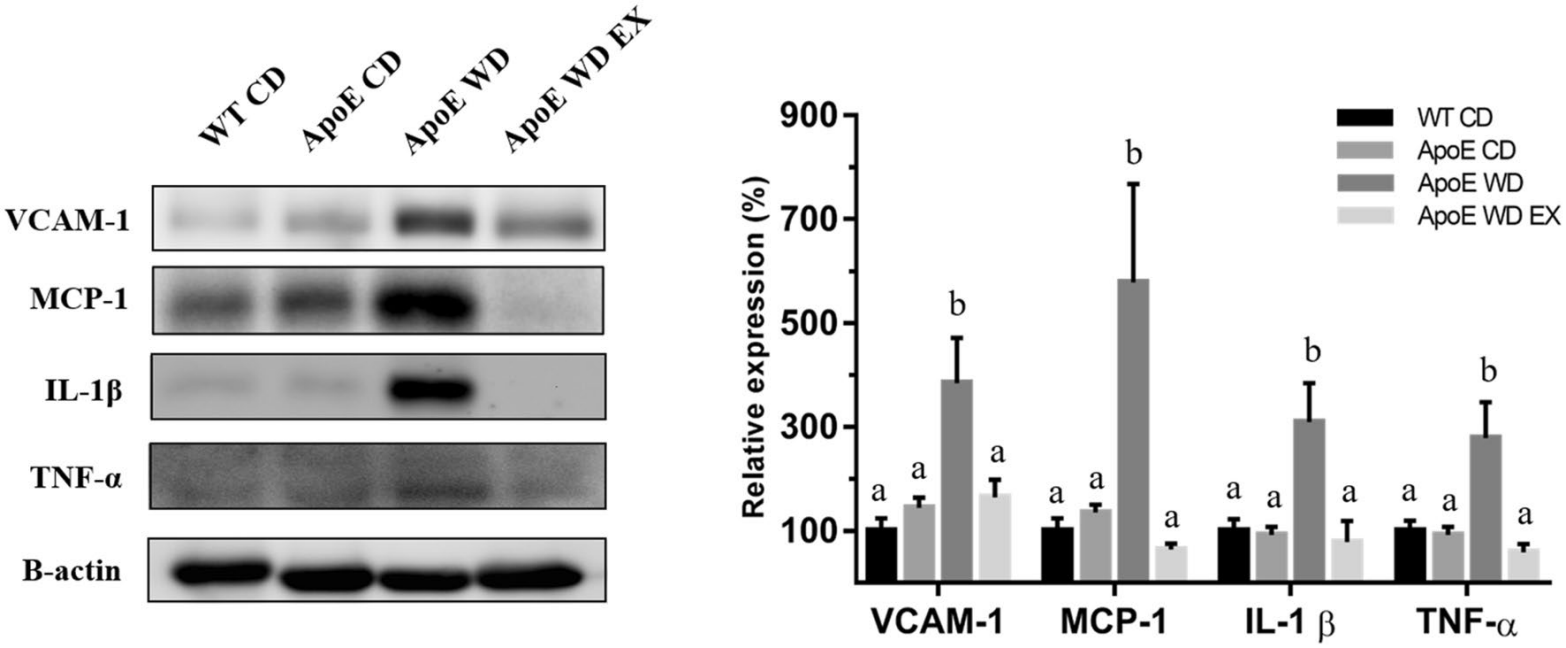
Effects of endurance exercise on aortic chemokine and proinflammatory cytokine expressions. VCAM-1, MCP-1, IL-1β, and TNF-α expression in the aorta was determined through Western blotting. Values are mean ± SEM (*n* = 6). Different superscript letters (a and b) indicate significant differences (*P* < 0.05) in one-way ANOVA.

Chemokines, from the largest family of cytokines, play critical roles in all stages of atherosclerotic development, in which they are prominently expressed in cells, such as endothelial and smooth muscle cells and leukocytes^26^. The chemokines and cytokines in aortic tissue were analyzed to determine the effects of exercise on atherosclerotic processes. Chemokine (VCAM-1 and MCP-1) and proinflammatory cytokine (IL-1β and TNF-α) expression in the aorta differed significantly among groups (F(3,20) = 6.72, *P* = 0.003; F(3,20) = 6.26, *P* = 0.004; F(3,20) = 5.2, *P* = 0.008; F(3,20) = 6.9, *P* = 0.002, respectively). No significant differences were noted between the WT CD and ApoE CD groups in the expression of VCAM-1, MCP-1, IL-1β, or TNF-α (Fig. 6). However, the ApoE WD group exhibited significantly higher VCAM-1, MCP-1, IL-1β, and TNF-α expression than did the chow diet groups (WT CD and ApoE CD). The highest VCAM-1 and MCP-1 expression were found in the ApoE WD group, which could mediate the adhesion and recruitment of leukocytes to the vascular endothelium for migration to inflamed site; VCAM-1 and MCP-1 were also reciprocally activated by the high levels of the aortic proinflammatory proteins IL-1β and TNF-α to cause more severe atherosclerotic lesions, which were observed through pathological examination. Exercise intervention in the ApoE WD EX group significantly lowered the expression of VCAM-1, MCP-1, IL-1β, and TNF-α to mitigate the chemoattraction of immune cells and local inflammation in the aorta, ameliorating atherosclerotic development; we also observed this as milder atherosclerotic lesions in the aortic root (Fig. 2).

## Discussion

CVD is the leading cause of death worldwide and can be categorized into coronary heart disease, cerebrovascular disease, peripheral artery disease, and aortic atherosclerosis. As economies have advanced, many jobs have shifted from being physically demanding to being sedentary, and this shift is often accompanied by longer working hours, which may explain the significant and steady increase in CVD incidence in past decades. Physical inactivity and poor diets (high in calories, saturated fats, and sugars) and lifestyle choices can cause obesity-associated metabolic syndromes (hyperglycemia, hyperlipidemia, and hypertension) associated with the development of atherosclerosis^22^. Exercise was determined to be an effective strategy for obesity management and CVD improvement through the regulation of mitochondrial function and myokine secretion, restoration and improvement of the vasculature, and mitigation of inflammation^23^. The microbiota influences a range of physiological processes, including host immunity, metabolism, and pathogenesis^24^, and homeostasis and physiological immunomodulation can be affected by environmental conditions and exercise stimuli^25^. In this study, we employed a murine model with ApoE knockout in combination with a WD to evaluate the effects of endurance exercise on atherosclerotic development. Twelve weeks of endurance exercise ameliorated obesity and atherosclerotic lesions through decreases in chemokines (VCAM-1 and MCP-1) and inflammatory cytokines (IL-1β and TNF-α) as well as the microbiota associated with inflammation (*Desulfovibrio*, *Tyzzerella*, and Lachnospiraceae_ge) and SCFA production (*Desulfovibrio*, *Tyzzerella*, and Lachnospiraceae_ge).

Atherosclerosis, a chronic inflammatory disease, is triggered by lipid retention in the arterial wall, and circulating lipoprotein particles, macrophages, and cholesterol promote plaque formation in the intima. The phagocytic activity of macrophages in lipoprotein ingestion causes the formation of foam cells with lipid droplets and dying macrophages, which constitute the necrotic core in plaques. Furthermore, plaque macrophages and their associated immune cells continually contribute to the local inflammatory response through the secretion of proinflammatory cytokines and chemokines and the offing application of oxidative stress during atherosclerosis^26^. The IL-1β and TNF-α secreted by macrophages, lymphocytes, natural killer cells, and vascular smooth muscle cells are proatherogenic cytokines. A genetic knockout of IL-1β was demonstrated by a significantly decrease atherosclerotic severity in the ApoE-deficient mice, and a simultaneous decrease in aortic VCAM-1 and MCP-1 was also observed^27^. The negative dominance of TNF-α decoy, a recombinant soluble TNF-αp55 receptor, can also ameliorate atherogenesis, endothelial adhesion, and elevation of inflammatory markers^28^. Besides, the VCAM-1 can be also upregulated the interaction between leukocytes and the endothelium with TNF-α activation^29^. Pharmaceuticals such as statins, aspirin, methotrexate, and colchicine may prevent or delay atherogenesis or CVD progression. However, regular exercise induced antiatherogenic effects through the amelioration of inflammation and immune cell chemotaxis in this study (Table 1 and Fig. 6).

The ApoE knockout mice were genetically modified to C57BL/6-*Apoe*^*em1Narl*^/Narl mice by using CRISPR/Cas9, and their phenotypes were manipulated with a high- cholesterol and high-fat WD to induce pathological atherosclerosis. ApoE knockout mice are widely used as an atherosensitive platform to evaluate the effect of modulator genes on the development and progression of atherosclerosis. The mechanisms of atherosclerotic regulation and therapeutic strategies were extensively investigated using an ApoE knockout model as a study platform^30^. LDL receptor and ApoE knockout in mice induced severe dyslipidemia and liver steatosis as well as atherosclerotic plaque formation; inflammatory cytokine levels and atherosclerotic syndrome were also exacerbated^31^. Our results demonstrated that ApoE knockout with a normal diet resulted in hypercholesterolemia and arterial thickening as compared with WT mice, and the WD aggravated the hyperlipidemia, inflammation, and atherosclerotic lesions in the ApoE knockout mice, as detailed in Table 1 and Fig. 1.

In a previous study of the effects of exercise on ApoE knockout model with a high-fat det, a 12-week swimming protocol (60 min/day) alleviated hyperlipidemia indexes and lipid metabolism through the activation of the *PPARγ*, *CPT1*, and *MCAD* genes^32^. However, the similar 12-week swimming intervention in our study did not affect lipid indexes, possibly because of our lower training volume (40 min/day); however, our chosen intensity ameliorated obesity and atherosclerosis. For physiological adaption, the chronic and acute exercise could contribute the anti-inflammation and pro-inflammation, but the inflammatory effect caused by the high-intensity, frequent, and prolonged exercise could accelerate severity in atherosclerotic athlete. The higher coronary artery calcification among athletes may not associated with an increased risk for cardiovascular events like the general population possible due to plaque stabilization with exercise training effects^33^. Therefore, the regular exercise and training volume could be important factors to alleviate the atherosclerosis. In clinical guideline of exercise to cardiovascular disease, the low to moderate endurance intensity, exercise frequency (> 150 min/week with at least 2 sections/week), resistant exercise and caloric expenditure (1000–2000 kcal/week) could also be considered as secondary prevention through exercise-based cardiac rehabilitation for cardiovascular health outcomes^34^.

The gut microbiota may be crucial to the integrity of the intestinal barrier and its effects on immune system development and immune mediator modulation^35^. Dysbiosis and gut barrier hyperpermeability can result in inflammation associated with diseases such as inflammatory bowel disease and colitis-associated cancers^36^. SCFAs, such as acetate, propionate, and butyrate, can affect intestinal barrier function, inflammatory regulation, oxidative stress, and carcinogenesis through the major signaling mechanisms of histone acetylation promotion and G-protein-coupled receptor activation^37^. In our study, dysbiosis resulted in a higher relative abundance of *Desulfovibrio*, *Parabacteroides*, *Bacteroides*, Ruminococcaceae_UCG_009, *Lactococcus*, Peptococcaceae_ge, *Ruminoclostridium_5*, Ruminococcaceae_ge, *Tyzzerella*, *and* Lachnospiraceae_ge. A correlation between significant enrichment of the Lachnospiraceae and Ruminococcaceae families and atherosclerotic lesions was also observed in another study employing ApoE knockout and a WD^38^. The plasma levels of triglycerides and cholesterol were positively correlated with *Tyzzerella* in a high-fat, high-fructose diet animal model^39^, and increased abundance of *Tyzzerella* is a risk factor for CVD^40^. *Desulfovibrio* spp. also exhibited significantly higher prevalence in patients with inflammatory bowel disease than in healthy individuals^41^. However, exercise intervention (ApoE WD EX) significantly decreased the abundance of *Desulfovibrio*, *Tyzzerella*, and Lachnospiraceae_ge and elevated the abundance of *Rikenella*, *Dubosiella*, and *Faecalibaculum* through fucoidan supplementation for the amelioration of cyclophosphamide-induced inflammation and for the intestinal integrity in relation to SCFA production^42^. Furthermore, lipocalin 2, an antimicrobial peptide expressed in the intestine, modulated obesity-associated and metabolic dysregulation through SCFA production regulated by *Dubosiella* and *Angelakisella*^43^. In addition, enriched *Dubosiella* in fermented rice bran–treated mice improved insulin sensitivity, antioxidative capacity, and lipid levels^44^. In another study, *Faecalibaculum* and its homology, *Holdemanella biformis*, demonstrated intestinal antitumorigenic effects through the production of SCFAs, which controlled the protein acetylation and tumor cell proliferation underlying calcineurin inhibition and NFATc3 activation^45^. SCFAs and their specific G-protein-coupled receptor 43 agonists also exhibited anti-inflammation and oxidative stress inhibition capacity^46^. Thus, the dysbiosis caused by a WD and hyperlipidemia resulted in intestinal hyperpermeability and inflammation alongside lower SCFA production, eventually contributing to the acceleration of atherosclerotic development in the ApoE WD group in our study. The divergence in the microbial composition in the ApoE WD group was corresponded to severe atherosclerotic lesion areas in the aortic root, further indicating the diet-dependent effects of the gut microbiota on atherosclerosis.

**Figure 7:**
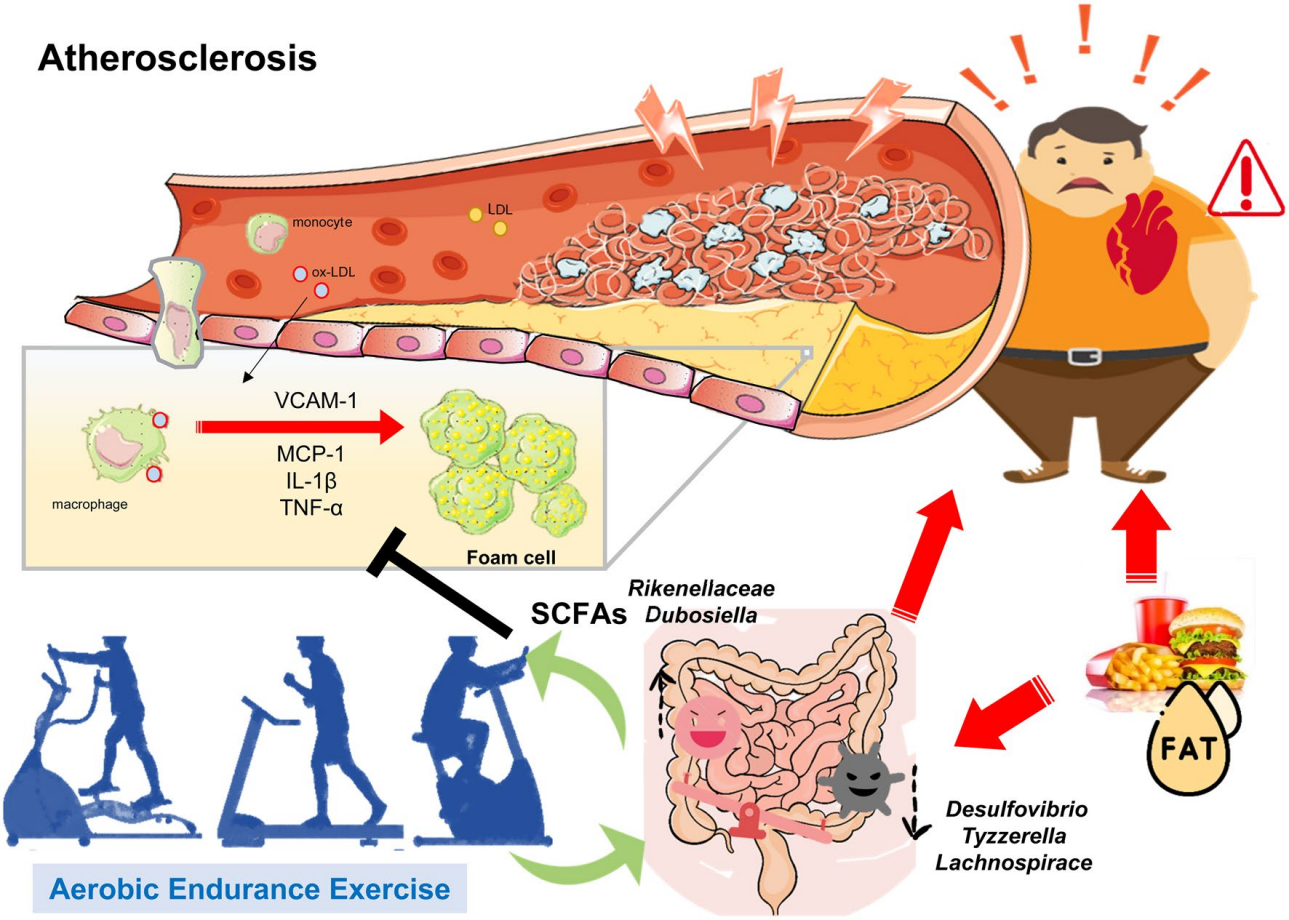
Proposed mechanism of endurance exercise–mediated modulation of microbiota and its metabolites in WD-induced atherosclerosis.

Moderate-intensity exercise assists with weight loss, fat loss, and cardiorespiratory fitness^47^ and can be medically prescribed for atherosclerosis^48^. Exercise can also induce physiological adaptations, including anti-inflammation and antioxidative stress activity, for disease prevention and therapy. The microbiota is a key modulator of health and disease progression, and exercise can affect the populations of beneficial and deleterious microbial species, microflora diversity, energy metabolism, and commensal bacteria growth^49^. Factors such as a high-fat diet could also alter the microbiota composition, reversing changes associated with obesity and preventing subsequent chronic disease development through the improvement of energy harvest and storage and reductions in gut permeability and inflammation^50^. *Desulfovibrio*, Ruminococcaceae, *Tyzzerella*, and Lachnospiraceae, associated with obesity-induced inflammation, dyslipidemia, and atherosclerosis, were significantly elevated in the ApoE WD group. In addition, the SCFAs produced by Rikenellaceae and *Dubosiella* were significantly elevated after exercise intervention. SCFAs can modulate immune and inflammatory responses through the activation of free fatty acid receptors 2 and 3 and the G-protein-coupled receptor 109A and the inhibition of histone deacetylases^51^. Therefore, the SCFAs produced by Rikenellaceae and *Dubosiella* in the ApoE WD EX group modulated chemotaxis through inflammation suppression in mild aortic atherosclerotic lesions. Overall, the data demonstrate that endurance exercise can modulate the microbiota to result in high SCFA production, alleviating WD-induced atherosclerotic progression through the inhibition of inflammation and the chemotaxis signaling pathway (Fig. 7).

## Conclusions

In summary, this study identified the mechanisms underlying the antiatherosclerotic effects of endurance exercise exerted by the microbial community and microbial-derived SCFAs. This is the first study to demonstrate that endurance exercise can modulate microbial populations, including those of *Desulfovibrio*, *Tyzzerella*, Lachnospiraceae_ge, Rikenellaceae, and *Dubosiella* for anti-inflammatory activity and SCFA production; aortic inflammatory responses significantly decreased after exercise with amelioration of atherosclerotic pathogenesis. 

## Supplementary Information

Supplementary Information.

## Data Availability

The gut microbiota sequencing datasets analyzed during the current study are available in the Gene Expression Omnibus (GEO) repository (Submission ID: SUB10967784 and BioProject ID: PRJNA798452), https://submit.ncbi.nlm.nih.gov/subs/bioproject/SUB10967784/.

## Abbreviations

ApoEKO: ApoE genetical knockout
CVDs: Cardiovascular diseases
HDL: High-density lipoprotein cholesterol
LDA: Linear discriminant analysis
LDL: Low-density lipoprotein cholesterol
LEfSe: Linear discriminant analysis effect size
MCP-1: Monocyte chemoattractant protein-1
PcoA: Principal co-ordinates analysis
SCFAs: Short chain fatty acids
TNF-α: Tumor necrosis factor alpha
VCAM-1: Vascular cell adhesion protein 1
WD: Western diet
DECIPHER: Database of Genomic Variation and Phenotype in Humans Using Ensemble Resources
