# Supplementary figures and images for: Endurance exercise ameliorates Western diet–induced atherosclerosis through modulation of microbiota and its metabolites
Source: Sci Rep. 2022 Mar 7;12:3612. doi: 10.1038/s41598-022-07317-x (PMC8901804; doi:10.1038/s41598-022-07317-x)

**Figure 6**

VCAM-1

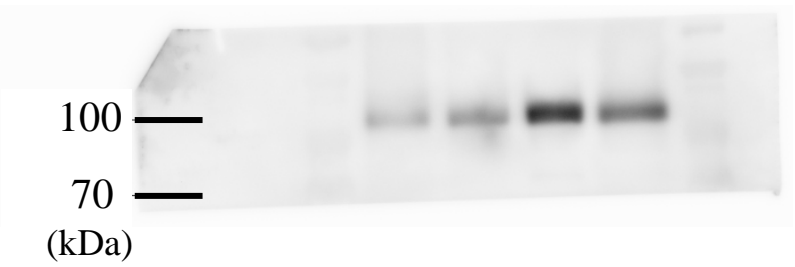

MCP-1

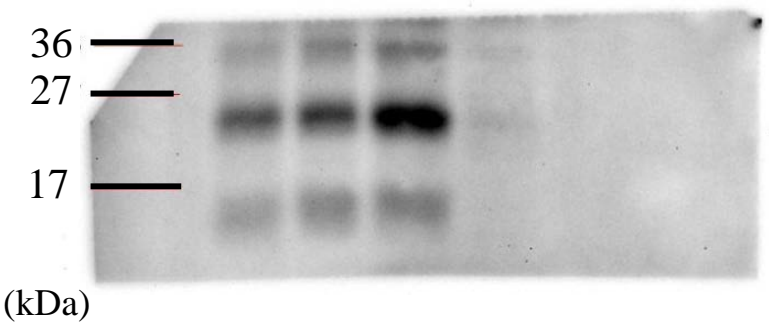

IL-1 beta

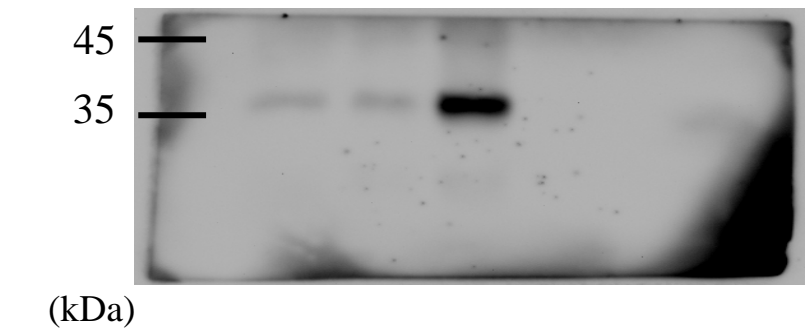

TNF- $\alpha$

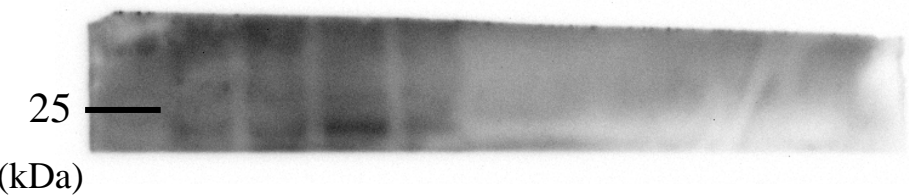

Beta-actin

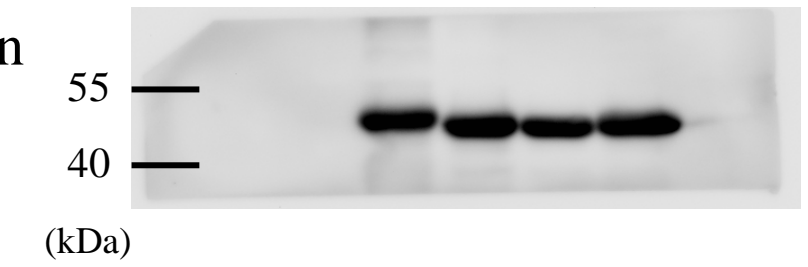

Supplement: Supplementary file 1 — Supplementary Information. [file 41598_2022_7317_MOESM1_ESM.pdf]
